# Current Challenges and Future Directions in the Multimodal Management of Gastric Cancer with Peritoneal Metastases

**DOI:** 10.3390/cancers18010105

**Published:** 2025-12-29

**Authors:** Andrea Cossu, Francesco Puccetti, Riccardo Rosati, Ugo Elmore

**Affiliations:** 1 Department of Gastrointestinal Surgery, IRCCS San Raffaele Scientific Institute, 20132 Milan, Italy; cossu.andrea@hsr.it (A.C.); rosati.riccardo@hsr.it (R.R.); elmore.ugo@hsr.it (U.E.); 2 School of Medicine, Vita-Salute San Raffaele University, 20132 Milan, Italy

**Keywords:** gastric cancer, peritoneal metastases, HIPEC, PIPAC, NIPS, chemotherapy

## Abstract

Peritoneal metastases represent one of the most dreadful manifestations of gastric cancer and continue to drive poor outcomes despite significant advances in systemic therapy. Accurate staging—beginning with laparoscopy—remains essential for avoiding non-beneficial surgery and ensuring appropriate allocation to systemic or locoregional treatment pathways. Although modern systemic agents, including immunotherapy and targeted therapies, have transformed the broader management of metastatic disease, their impact in the peritoneal compartment remains limited, reflecting its unique biological and pharmacokinetic constraints. Locoregional approaches such as CRS–HIPEC, PIPAC, and NIPS have expanded the therapeutic armamentarium and have shown encouraging signals in selected populations. Recent randomized studies, including ESTOK01 and PERISCOPE II, emphasize the importance of careful patient selection, technical standardization, and optimal sequencing, while ongoing trials—such as PREVENT, GASTRICHIP, and CONVERGENCE—seek to refine the integration of systemic and intraperitoneal strategies. Yet the field continues to advance without the benefit of validated predictive biomarkers capable of guiding therapeutic decisions. This limitation constrains clinical progress and underscores the need for a stronger translational framework. Future improvement in the management of gastric cancer with peritoneal metastases will depend on the identification of robust biological predictors of response, enabling more rational patient selection and the development of truly personalized multimodal approaches.

## Simple Summary

Peritoneal metastases from gastric cancer represent one of the most challenging disease presentations, often leading to limited survival and a rapid decline in quality of life. Although modern treatments such as chemotherapy, immunotherapy and targeted therapies have improved outcomes in metastatic gastric cancer overall, they are far less effective when the disease involves the peritoneal cavity. Correct treatment planning must therefore begin with accurate disease assessment, including staging laparoscopy, which can detect hidden peritoneal spread and help avoid unnecessary major surgery. Several innovative locoregional approaches have shown encouraging results in selected patients, but they are not yet fully validated for widespread adoption. Most importantly, we still lack reliable biological indicators to guide treatment selection and match the right approach to the right patient. Continued research is crucial to develop more personalized strategies and improve care for individuals with gastric cancer complicated by peritoneal metastases.

## 1. Editorial

Peritoneal involvement remains a common and clinically significant pattern of disease spread in gastric cancer. Its presence profoundly influences prognosis, treatment selection, and overall management strategies. While advances in systemic therapy have reshaped the therapeutic landscape of advanced gastric cancer, peritoneal disease continues to pose distinct clinical challenges. These considerations have stimulated growing interest in multimodal treatment approaches and in refining patient selection through improved staging and emerging translational insights.

### 1.1. Why Peritoneal Metastases Still Matter

Peritoneal metastases (PMs) represent one of the most aggressive and therapeutically challenging manifestations of gastric cancer. Their rapid dissemination, intrinsic resistance to systemic therapy, and unfavorable microenvironment continue to drive early treatment failure and poor survival. Despite advances in systemic therapy, the peritoneal compartment behaves as a distinct biological niche—characterized by limited vascularization, pharmacokinetic barriers, and stromal resistance—that reduces therapeutic penetration and efficacy [1]. This fundamental divergence underscores why PM cannot be approached as a simple extension of metastatic disease elsewhere and why dedicated multimodal strategies remain essential.

### 1.2. Staging as the First Therapeutic Decision

Accurate staging is the true starting point of care for patients with locally advanced or high-risk gastric cancer. Staging laparoscopy remains the most sensitive method to detect radiologically occult PM and consistently prevents non-therapeutic gastrectomies and inappropriate treatment pathways [2]. Peritoneal cytology further refines prognostication, with positive cytology conveying outcomes similar to overt carcinomatosis and increasingly recognized as metastatic disease in major guidelines [3,4,5]. In this sense, staging is not merely diagnostic: it is a therapeutic decision point that directs patients toward systemic treatment, surgery, or integration of locoregional modalities.

At the same time, staging laparoscopy is increasingly recognized not only as a diagnostic tool but also as an opportunity for biological sampling. Emerging molecular analyses of peritoneal fluid and metastases are beginning to offer deeper insight into disease behavior and may eventually contribute to more refined, biology-driven criteria for selecting candidates for intraperitoneal therapy.

### 1.3. Systemic Therapy Alone Is Not Enough

Systemic therapy for metastatic gastric cancer has advanced substantially with perioperative FLOT [6], nivolumab combined with chemotherapy [7], and emerging targeted agents such as pembrolizumab–trastuzumab [8], trastuzumab–deruxtecan [9], zolbetuximab [10], bemarituzumab [11], and durvalumab [12]. These developments have shown improved outcomes in biomarker-selected populations. Yet their benefits translate only marginally to the peritoneal compartment. The RENAISSANCE (IKF-575) trial [13] highlighted this disconnect: patients with PM demonstrated inferior outcomes despite disease control after induction therapy, confirming that metastatic patterns are not interchangeable and that therapeutic paradigms effective in other sites may not apply to the peritoneum; however, the results of this trial are biased by high incidence of perioperative complications.

### 1.4. Understanding the Role of CRS–HIPEC Today

CRS–HIPEC has a long-standing rationale supported by retrospective studies and large multicenter datasets such as CYTO-CHIP [14] as well as national surgical experiences [15]. Earlier randomized trials, including GASTRIPEC-I [16] and the study by Yang et al. [17], suggested a potential benefit in highly selected patients, particularly those with limited peritoneal burden (PCI < 7).

More recently, PERISCOPE II [18] offered a more nuanced perspective. The trial did not demonstrate a survival advantage for CRS–HIPEC over continued systemic therapy after induction. Interpretation, however, requires caution. The HIPEC protocol employed a 30 min oxaliplatin perfusion and normothermic docetaxel—parameters that diverge from widely used contemporary standards and may have reduced cytotoxic efficacy. Additionally, the study was prematurely closed due to increased severe adverse events in the CRS–HIPEC arm, an outcome not consistently reported by high-volume centers for peritoneal disease internationally. These considerations highlight the need for technical standardization, rigorous selection, and integration with systemic therapy when evaluating CRS–HIPEC in modern multimodal pathways.

### 1.5. Evolving Evidence from Ongoing Multimodal Trials

Among ongoing studies, GASTRICHIP occupies a unique position, although its design reflects the era in which it was conceived. The protocol employs oxaliplatin-based HIPEC perfused for only 30 min—parameters now considered outdated—and relies on a five-year overall survival endpoint that delays data maturity [19]. Given the rapid evolution of systemic and intraperitoneal therapies, final results may be available in a different therapeutic landscape from that of the study’s inception. These factors illustrate the inherent difficulty of conducting long-term perioperative trials in a rapidly changing multimodal context.

In contrast, PREVENT/FLOT-9 represents a more contemporary and pragmatic effort. By integrating perioperative intraperitoneal chemotherapy into current systemic regimens and adopting a two-year disease-free survival endpoint, it is better positioned to generate timely and clinically relevant evidence aligned with current knowledge of peritoneal relapse biology [20].

A more modern perspective is offered by CONVERGENCE, currently in activation and presented as a study concept at PSOGI Congress 2025 [21], a trial designed to assess the value of CRS–HIPEC after a diversified induction phase. Patients first receive systemic therapy, which may include chemotherapy, immunotherapy, targeted agents and, in selected centers, bidirectional approaches combining systemic treatment with intraperitoneal strategies such as NIPS or PIPAC. Only those achieving disease control proceed to randomization between CRS–HIPEC and continuation of systemic therapy. Rather than comparing multiple locoregional modalities, CONVERGENCE tests whether CRS–HIPEC adds benefit when applied selectively after a biologically meaningful induction phase. This response-based design reflects current multimodal practice and may help clarify the role of CRS–HIPEC within contemporary treatment sequences for gastric cancer with peritoneal metastases.

### 1.6. Where PIPAC Stands Now

PIPAC has emerged as a minimally invasive and repeatable locoregional treatment, offering advantageous peritoneal drug distribution with limited systemic exposure. Its feasibility and symptom control have led to inclusion in the AIOM (Italian Association of Medical Oncology) national guidelines as a palliative option for unresectable or refractory PM but currently restricted to clinical trial settings [22].

The randomized ESTOK01 trial [23], conducted in patients with highly advanced disease, did not demonstrate survival benefits and was halted early due to safety concerns. Nevertheless, prospective institutional series—including those by Alyami and colleagues [24]—suggest that earlier integration of PIPAC, particularly when combined with systemic therapy, may induce disease stabilization in selected patients. Another perspective currently under exploration is offered by the VEROne study [25], which seeks to define the potential role of neoadjuvant PIPAC in patients with limited peritoneal disease or in prophylactic settings. Although definitive evidence is still awaited, this trial reflects the growing interest in earlier and more strategic integration of intraperitoneal therapy.

### 1.7. NIPS: Current Evidence and Rationale

Neoadjuvant intraperitoneal and systemic chemotherapy (NIPS) emerged from the Eastern oncologic framework, which has long emphasized strategies targeting peritoneal dissemination to intensify local drug exposure in patients at risk of peritoneal dissemination. Its rationale is based on repeated intraperitoneal administration of paclitaxel combined with systemic therapy, attempting to overcome the pharmacokinetic barriers of the peritoneal cavity. Within this framework, the PHOENIX trial [26], though not meeting its primary endpoint, produced clinically meaningful signals—particularly in patients with ascites—supporting the biological rationale for sustained intraperitoneal taxane exposure.

The INPACT trial [27] builds on this concept by evaluating NIPS in patients with low-volume peritoneal disease or positive cytology, aiming to clarify whether repeated intraperitoneal paclitaxel can enhance outcomes compared with systemic therapy alone. While the trial adopts a clinically meaningful endpoint—two-year overall survival—the superiority of intraperitoneal over intravenous paclitaxel has not yet been established, underscoring the need for further prospective data to refine patient selection, timing and integration within multimodal pathways.

### 1.8. The Urgency of Translational Research

Despite increasing therapeutic options, the absence of validated predictive biomarkers remains the major unmet need. PCI and radiologic evaluation insufficiently capture the molecular heterogeneity and biological diversity of peritoneal disease. Novel translational tools—including molecular cytology [28], peritoneal ctDNA [29], exosome-derived signatures [30], and patient-derived organoids—offer promising avenues to refine risk stratification, anticipate treatment response, and guide real-time decision-making. Their incorporation into clinical trial design is essential to advance toward truly biologically informed multimodal strategies.

## 2. Conclusions

Peritoneal metastases remain one of the greatest therapeutic challenges in gastric cancer. Despite advances in systemic therapy, including immunotherapy and next-generation targeted agents, their unique biology and pharmacokinetic barriers continue to limit therapeutic efficacy. Locoregional strategies such as CRS–HIPEC, PIPAC, and NIPS offer promising—but still incompletely validated—options for selected patients. Recent randomized trials highlight the need for rigorous selection, technical consistency and appropriate treatment timing.

The future of care for gastric cancer with peritoneal metastases will depend on integrating translational research with clinical decision-making, with predictive biomarkers playing a pivotal role in optimizing patient selection and personalizing multimodal strategies.

## Data Availability

Data are available upon request.
